# Missed opportunities of inclusion in a cohort of HIV-infected children to initiate antiretroviral treatment before the age of two in West Africa, 2011 to 2013

**DOI:** 10.7448/IAS.19.1.20601

**Published:** 2016-03-23

**Authors:** Désiré L Dahourou, Madeleine Amorissani-Folquet, Malik Coulibaly, Divine Avit-Edi, Nicolas Meda, Marguerite Timite-Konan, Vic Arendt, Diarra Ye, Clarisse Amani-Bosse, Roger Salamon, Philippe Lepage, Valériane Leroy, for the Monod Anrs 12206 Study Group

**Affiliations:** 1 MONOD Project, ANRS 12206, Centre de Recherche Internationale pour la Santé, Ouagadougou, Burkina Faso; 2 Inserm, U1219, Institut de Santé Publique, Epidémiologie et Développement, University of Bordeaux, Bordeaux, France; 3 Centre Muraz, Bobo-Dioulasso, Burkina Faso; 4 PACCI Programme, Site ANRS, Projet Monod, Abidjan, Côte d'Ivoire; 5 Pediatric Department, CHU of Cocody, Abidjan, Côte d'Ivoire; 6 University of Ouagadougou, Ouagadougou, Burkina Faso; 7 Pediatric Department, Centre Hospitalier Universitaire (CHU) de Yopougon, Abidjan, Côte d'Ivoire; 8 Department of Infection and Immunity, Luxembourg Institute of Health, Luxembourg; 9 Pediatric Department, CHU Charles de Gaulle, Ouagadougou, Burkina Faso; 10 Pediatric Department, Hôpital Universitaire des Enfants de la Reine Fabiola, Université Libre de Bruxelles, Brussels, Belgium; 11 Inserm, U1027, Université Toulouse, Toulouse, France

**Keywords:** children, West Africa, HIV, acceptability, early antiretroviral therapy, linkage to care, access to care

## Abstract

**Introduction:**

The World Health Organization (WHO) 2010 guidelines recommended to treat all HIV-infected children less than two years of age. We described the inclusion process and its correlates of HIV-infected children initiated on early antiretroviral therapy (EART) at less than two years of age in Abidjan, Côte d'Ivoire, and Ouagadougou, Burkina Faso.

**Methods:**

All children with HIV-1 infection confirmed with a DNA PCR test of a blood sample, aged less than two years, living at a distance less than two hours from the centres and whose parents (or mother if she was the only legal guardian or the legal caregiver if parents were not alive) agreed to participate in the MONOD ANRS 12206 project were included in a cohort to receive EART based on lopinavir/r. We used logistic regression to identify correlates of inclusion.

**Results:**

Among the 217 children screened and referred to the MONOD centres, 161 (74%) were included and initiated on EART. The main reasons of non-inclusion were fear of father's refusal (48%), mortality (24%), false-positive HIV infection test (16%) and other ineligibility reasons (12%). Having previously disclosed the child's and mother's HIV status to the father (adjusted odds ratio (aOR): 3.20; 95% confidence interval (95% CI): 1.55 to 6.69) and being older than 12 months (aOR: 2.05; 95% CI: 1.02 to 4.12) were correlates of EART initiation. At EART initiation, the median age was 13.5 months, 70% had reached WHO Stage 3/4 and 57% had a severe immune deficiency.

**Conclusions:**

Fear of stigmatization by the father and early competing mortality were the major reasons for missed opportunities of EART initiation. There is an urgent need to involve fathers in the care of their HIV-exposed children and to promote early infant diagnosis to improve their future access to EART and survival.

## Introduction

The paediatric epidemic of HIV remains a major concern in the world. In 2012, three million children were living with HIV infection worldwide, 91% of whom were in sub-Saharan Africa [1]. Despite a decrease in HIV prevalence among pregnant women over the last decade, the epidemic is still widespread in Burkina Faso (1.7% prevalence in 2011 [2]) and in Côte d'Ivoire (4.5% prevalence in 2009 [3]). Although the coverage of prevention of mother-to-child transmission (PMTCT) programmes has improved in both countries, it still remains insufficient. In 2011, 67% and 29% of HIV-positive women received the PMTCT intervention in Burkina Faso [2] and Côte d'Ivoire [4], respectively, with a growing HIV paediatric epidemic. In the absence of any intervention, the mortality of HIV-infected children remains high, reaching 50% at the age of two [5].

In 2008, the antiretroviral CHER trial demonstrated the efficacy of starting early antiretroviral therapy (EART) before two months of age in reducing infant mortality in HIV-infected infants compared with those treated according to the World Health Organization (WHO) 2006 recommendations: 4% versus 16% respectively [6]. Since then, the WHO has recommended the initiation of EART in all HIV-infected children before the age of 12 months, regardless of their disease progression [7], extended up to 24 months of age in 2010 [8]. Consequently, a systematic early infant diagnosis of HIV was recommended for all HIV-exposed children from the age of six weeks [8] in order to initiate EART in those infected by HIV.

However, access to EART for HIV-infected children is complex in the African context as operational issues further complicate the situation. Overall, access to antiretroviral therapy (ART) has significantly improved, but remains insufficient, with only 34% of eligible children in sub-Saharan Africa having access to treatment in 2012, compared to 64% of adults [1]. First, access to early infant diagnosis under the age of 18 months requires sophisticated PCR techniques due to the persistence of maternal antibodies in the child's blood. These techniques are not available everywhere [9]. Second among the barriers, the lack of service delivery and low acceptability by healthcare workers and the community are frequent [10–12]. Consequently, providing a continuum of care for HIV-exposed children remains challenging. Those HIV-infected children who have a chance to access ART are frequently treated late, at an advanced age (median age greater than five years) and at an advanced stage of the disease [13,14].

Since the promulgation of the WHO 2010 recommendations, no study has assessed the missed opportunities of the pre-EART cascade beyond HIV diagnosis and the acceptability of EART proposed in HIV-infected children less than two years old in West Africa. We studied the inclusion process and the family acceptability of EART as part of the pre-ART cascade and its correlates among HIV-infected children less than two years old in Abidjan and Ouagadougou through the description of the inclusion process in the therapeutic cohort of the MONOD ANRS 12206 project.

## Methods

### Settings

The MONOD ANRS 12206 project was funded by the French National Agency for Research on AIDS and Viral Hepatitis (ANRS), the European and Developing Countries Trials Partnership (EDCTP) and the Luxembourg Institute of Health. This was a multicentre project launched in two West African countries (Burkina Faso and Côte d'Ivoire) targeted at EART implementation before the age of two as recommended by the WHO in 2010. This project was conducted in the two paediatric wards of the Yalgado Ouédraogo and Charles de Gaulle University Hospitals in Ouagadougou, Burkina Faso, and in two public paediatric HIV programme sites (FSU Abobo-Avocatier, CEPREF-Yopougon) as well as in the two paediatric wards of the Yopougon and Cocody University Hospitals in Abidjan, Côte d'Ivoire.

### Study design

We studied the inclusion process of children screened to be enrolled in the MONOD ANRS 12206 project until their confirmed inclusion with EART initiation. Eligible children were initially included in a 12-month therapeutic prospective cohort with a twice-daily EART combination (zidovudine (AZT) or abacavir (ABC))+lamivudine (3TC)+lopinavir/ritonavir (LPV/r) in addition to cotrimoxazole prophylaxis and therapeutic education (teaching the caregiver how to comply with the daily dosages and hours of treatment).

### Participants

All HIV-1-infected children screened by HIV DNA PCR on dried blood spot samples under the umbrella of the national screening programme in urban centres, less than two years old and ART-naive except for PMTCT prophylaxis of HIV were referred with their parents (or the legal caregiver if parents were not alive) to one of the MONOD healthcare facilities in Abidjan and Ouagadougou to be pre-included. Those children with HIV infection confirmed by DNA PCR on blood sample, with haemoglobin levels greater than 7 g/dl; neutrophils greater than 750/mm^3^; creatinine five times below the normal threshold; aspartate aminotransferase (AST) and alanine-amino-transferase (ALT) five times below the normal threshold and whose parents (or mother if she was the only legal guardian, or the legal caregiver if parents were not alive) consented to participation in the MONOD ANRS 12206 project, were initiated on EART. Children diagnosed with tuberculosis at pre-inclusion were included in a separate cohort and received an efavirenz (EFV)-based therapy because of the drug interaction between LPV/r and tuberculosis treatment [15].

### Data collection

Patients’ data were collected prospectively using standardized questionnaires at their first contact, pre-inclusion and inclusion visits. The socio-demographic data of mothers and children and PMTCT exposure data were collected at the first contact visit. After obtaining the parents’ written informed consent to participate in the project, all socio-demographic data, data related to the father's HIV status, DNA PCR to confirm the child's HIV status, haematology, biochemistry, CD4 percentage and viral load data of the children were collected at the pre-inclusion visit (four weeks before inclusion). At the inclusion visit, children's anthropometric and clinical data were collected. Clinical stage and immunodeficiency were defined according to the WHO recommendations issued in 2006 [16].

### Statistical analysis

We described the pre-ART inclusion process of children initially diagnosed as HIV infected and the causes of non-inclusion. We described the socio-demographic characteristics of the children at their first contact and pre-inclusion visits according to their inclusion in the EART therapeutic cohort. For children with a confirmed HIV infection status, we analyzed the correlates of inclusion in the therapeutic cohort, using a multivariate logistic regression including all uncorrelated variables associated in the univariate analysis with *p*<0.25. Then, we conducted a stepwise descending adjusted analysis.

Baseline categorical data are presented as frequencies (percentage), whereas continuous variables are presented using the mean and standard deviation (SD) for normally distributed continuous data or using median and interquartile ranges (IQR) for non-normally distributed continuous data. We compared categorical data using the chi-square test or Fisher's exact test, and we used Student's t-test or Wilcoxon rank-sum test to compare continuous data.

Weight-for-height, weight-for-age and height-for-age were used to assess children's nutritional status. Each outcome was standardized (z-score) for age and gender using the WHO growth standards [17]. We defined underweight at baseline as weight-for-age z-score (WAZ) <−2 SD and stunting as height-for-age z-score (HAZ) <−2 SD.

All statistical analyses were performed with SAS 9.3 (SAS Institute Inc., Cary, NC, USA) except for anthropometric indicators, which were analyzed with ENA/Epi Info 3.5.1.

### Ethical considerations

Both parents (or the mother if she was the only legal guardian, or the legal caregiver if parents were not alive) of each child enrolled gave their written informed consent to participate in the MONOD ANRS 12206 project, which was approved by both national ethics committees of Burkina Faso and Côte d'Ivoire. The consent had to be signed no later than the pre-inclusion visit.

## Results

### Cohort profile

**Figure 1:**
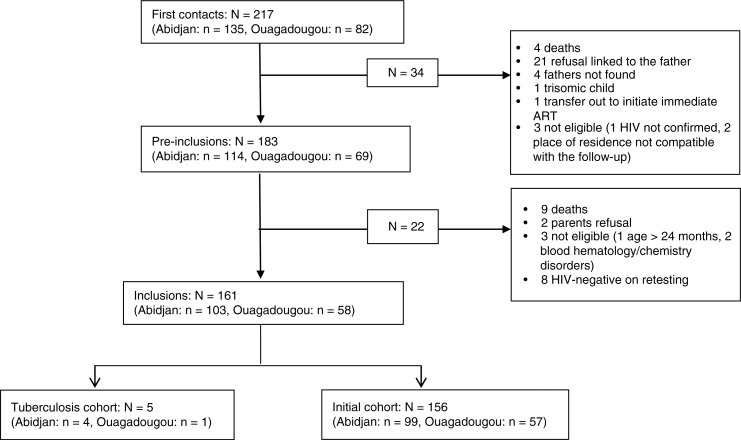
Cohort profile ofthe ANRS 12206 MONOD study, Abidjan, Ouagadougou, May 2011 to February 2013.

From May 2011 to February 2013, 217 children had a first contact visit, and of these 161 were included in the MONOD cohort (156 in the initial cohort and 5 in the tuberculosis cohort). The cumulative rate of inclusion was 74%. The main reasons for non-inclusion (*N=*56) were linked to the refusal of EART for the child by the father or father's denial of child's HIV status (27/56; 48%), early mortality (13/56; 24%), child found to be HIV negative on retested sample (9/56; 16%) and ineligibility for geographical reasons or due to severe disorders of baseline blood haematology/chemistry (7/56; 12%). Thus, among the 208 HIV-infected children confirmed eligible for treatment, 77% were initiated on EART (95% confidence interval (95% CI): 71% to 83%) (Figure 1).

### Characteristics of eligible children according to their inclusion in the EART therapeutic cohort (N=208)

Table 1 provides the baseline characteristics of the children according to their inclusion in the MONOD cohort. Overall, the median age at first HIV diagnosis and at the first contact visit were 8.4 months (IQR: 2.9 to 15.6) and 11.7 months (IQR: 6.0 to 17.3), respectively; 54% were girls. According to inclusion in the MONOD cohort, there was no difference in median age at first contact. However, median age at first HIV diagnosis was significantly different between those included and non-included children (*p*=0.03): children included were three months older at first HIV diagnosis than those not included. At the first contact visit, children were most often accompanied by both parents (56.7%); this proportion was significantly higher in children included (*p*<0.01). The mothers of the children included were more often single mothers compared to those of children not included (*p*=0.02). Overall, 62% of fathers had already been informed of the HIV status of their own child and of the mother; this proportion was significantly higher in children included (*p*<0.01).

**Table 1:** Characteristics of eligible children according to their inclusion in the ANRS 12206 MONOD study, Abidjan, Ouagadougou, May 2011 to February 2013

	Total *n*=208	Not included *n*=47	Included *n*=161	
		
Characteristics	*n* (%)	*n* (%)	*n* (%)	*p*
Median child's age (in months) at first contact (IQR)	11.9 (6.4 to 17.3)	9.5 (5.7 to 17.0)	12.9 (6.6 to 17.3)	0.17*
Child's age at first contact (in months)				0.08
<12	105 (50.5)	29 (61.7)	76 (47.2)	
≥12	103 (49.5)	18 (38.3)	85 (52.8)	
Median child's age (in months) at first HIV diagnosis (IQR)	8.4 (2.9 to 15.6)	5.9 (2.4 to 13.0)	9.0 (3.4 to 16.0)	0.03*
Age at first HIV diagnosis (in months)				0.07
<2	29 (14.1)	10 (22.2)	19 (11.8)	
≥2	177 (85.9)	35 (77.8)	142 (88.2)	
Gender				0.87
Male	95 (45.7)	21 (44.7)	74 (46.0)	
Female	113 (54.3)	26 (55.3)	87 (54.0)	
Vital status of the father				<0.01
Alive	146 (70.2)	11 (23.4)	135 (83.8)	
Deceased	10 (4.8)	1 (2.1)	9 (5.6)	
Unknown/missing	52 (25.0)	35 (74.5)	17 (10.6)	
Person accompanying child at the first contact				<0.01**
Mother alone	80 (38.5)	38 (80.8)	42 (26.1)	
Father alone	3 (1.4)	0 (0.0)	3 (1.9)	
Both parents	118 (56.7)	8 (17.0)	110 (68.4)	
Legal guardian	2 (1.0)	0 (0.0)	2 (1.2)	
Other	5 (2.4)	1 (2.2)	4 (2.4)	
Mother still in touch with the child's father				0.02**
Yes	175 (84.1)	46 (97.9)	129 (80.1)	
No	19 (9.1)	0 (0.0)	19 (11.8)	
Deceased	10 (4.8)	1 (1.8)	9 (5.6)	
Unknown	4 (2.0)	0 (2.1)	4 (2.5)	
Father informed of the mother's HIV status (*N*=198***)				<0.01
Yes	124 (62.6)	20 (43.5)	104 (68.4)	
No	66 (33.3)	25 (54.3)	41 (27.0)	
Unknown	8 (4.1)	1 (2.2)	7 (4.6)	
Father informed of his child's HIV status (*N*=198***)				<0.01**
Yes	123 (62.1)	20 (43.5)	103 (67.8)	
No	74 (37.4)	26 (56.5)	48 (31.5)	
Unknown	1 (0.51)	0 (2.1)	1 (0.7)	

IQR: interquartile range

* *p*-value from Wilcoxon rank-sum test

** *p*-value from Fisher's exact test

*** excluding data for children whose fathers were deceased.

Correlates of inclusion were investigated in a multivariate analysis (Table 2). Children who were accompanied by both parents at the first contact visit were 10 times more likely to be included and initiated on EART. In the final adjusted analysis, we found that children were more likely to be included when they were older than 12 months at the first contact visit (adjusted odds ratio (aOR): 2.05; 95% CI: 1.02 to 4.12) and when the father was informed about the HIV status of both the mother and the child (aOR: 3.20; 95% CI: 1.55 to 6.69) (Table 2).

**Table 2:** Factors associated with inclusion in the ANRS 12206 MONOD study, Abidjan, Ouagadougou, May 2011 to February 2013 (*N*=208)

	Unadjusted bivariate analysis		Adjusted multivariate analysis	
		
Characteristics	OR	95% CI	OR	95% CI
Child's age at first contact (months)				
<12	Reference		Reference	
≥12	1.80	0.92 to 3.50	2.05	1.02 to 4.12
Child's age at first HIV diagnosis (months)				
<2	Reference			
≥2	2.13	0.91 to 4.99		
Gender				
Male	Reference			
Female	0.95	0.49 to 1.82		
Person accompanying child at the first contact				
Mother or legal guardian	Reference			
Both parents or other	10.24	4.59 to 22.84		
Father informed of HIV status of the mother and/or his child				
No/unknown	Reference		Reference	
Mother and child	3.04	1.50 to 6.19	3.20	1.55 to 6.59
Mother only	5.18	1.10 to 24.34	6.21	1.29 to 29.78
Child only	3.96	0.82 to 19.04	4.08	0.83 to 19.89

OR: odds ratio; CI: confidence interval.

### Baseline variables of children included according to study sites

The baseline characteristics of the 161 children included in the MONOD cohort are described in Table 3 according to their study site. The median age at ART initiation of children enrolled was 13.7 months (IQR: 7.7 to 18.4). This age was significantly different between the two study sites (*p*=0.03): children in Ouagadougou were two months older than those in Abidjan. The children were female (54%) and lived with both parents (67%) regardless of the study sites. Overall, the main caregivers were mothers (83%). However, more fathers in Ouagadougou (26%) than in Abidjan (1%) were the main caregivers (*p=*0.01). In Abidjan, parents were significantly more likely to have access to tap water, electricity and refrigerators at home compared to those in Ouagadougou. Over half (51%) of the mothers did not receive any prenatal PMTCT intervention, and only 35% had ongoing ART at the time of inclusion of their child. Over half (51%) of the children included had not received any postnatal prophylaxis for PMTCT. Self-reported father's HIV test was unknown for 14% of the children. Only 64% of the fathers reported having taken an HIV test, but this proportion was significantly higher in Abidjan than in Ouagadougou: 68% versus 57%, respectively (*p*=0.02). Among the fathers who underwent screening for HIV infection, 40% reported being HIV positive, of whom 51% were on ART.

**Table 3:** Baseline socio-demographic characteristics of the 161 HIV-infected children included in the ANRS 12206 MONOD study according to site (Abidjan, Ouagadougou, May 2011 to February 2013)

	Total *n=*161	Abidjan *n*=103	Ouagadougou *n*=58	
		
Characteristics	*n* (%)	*n* (%)	*n* (%)	*p*
Median child's age (in months) at ART initiation (IQR)	13.6 (7.7 to 18.4)	12.9 (6.7 to 18.3)	14.6 (10.1 to 19.5)	0.03
Child's age at EART initiation (in months)				
<12	70 (43.5)	50 (48.5)	20 (34.5)	0.08
≥12	91 (56.5)	53 (51.5)	38 (65.5)	
Gender				0.38
Male	74 (46.0)	50 (48.5)	24 (41.4)	
Female	87 (54.0)	53 (51.5)	34 (58.1)	
Main caregiver				<0.01
Mother	133 (82.6)	93 (90.3)	40 (69.0)	
Father	16 (9.9)	1 (1.0)	15 (25.8)	
Other	12 (7.5)	9 (8.7)	3 (5.2)	
Type of housing				0.02
Individual housing	83 (51.5)	46 (44.7)	37 (63.8)	
Shared housing	78 (48.5)	57 (55.3)	21 (36.2)	
Tap water at home				<0.01
Yes	108 (67.1)	87 (84.5)	21 (36.2)	
No	53 (32.9)	16 (15.5)	37 (63.8)	
Electricity at home				<0.01
Yes	120 (74.5)	98 (95.2)	22 (37.9)	
No	41 (25.5)	5 (4.8)	36 (62.1)	
Fridge at home				0.02
Yes	36 (22.4)	29 (28.2)	7 (12.1)	
No	125 (77.6)	74 (71.8)	51 (87.9)	
Orphan				0.17
Both parents alive	146 (90.7)	91 (88.4)	55 (94.8)	
Mother deceased	6 (3.7)	6 (5.8)	0 (0.0)	
Father deceased	9 (5.6)	6 (5.8)	3 (5.2)	
Family structure				0.65
Two parents	109 (67.3)	71 (68.9)	38 (65.5)	
Single parent/missing	52 (32.1)	32 (31.1)	20 (34.5)	
Mother on ART***				0.06
Yes	56 (34.8)	42 (40.8)	14 (24.1)	
No	94 (58.4)	53 (51.4)	41 (70.7)	
Unknown	11 (6.8)	8 (7.8)	3 (5.2)	
Maternal PMTCT prophylaxis				0.79
Yes	66 (41.0)	41 (39.8)	25 (43.1)	
No	83 (51.5)	55 (53.4)	28 (48.3)	
Unknown	12 (7.5)	7 (6.8)	5 (8.6)	
Postnatal PMTCT prophylaxis				0.93
Yes	66 (41.0)	43 (41.7)	23 (39.7)	
No	83 (51.5)	52 (50.5)	31 (53.4)	
Unknown	12 (7.5)	8 (7.8)	4 (6.9)	
Father tested for HIV				0.03
Yes	103 (64.0)	70 (68.0)	33 (56.9)	
No	35 (21.7)	24 (23.3)	11 (19.0)	
Unknown	23 (14.3)	9 (8.7)	14 (24.1)	
Father HIV positive*** (*N*=103)				0.06**
Yes	41 (39.8)	29 (41.4)	12 (36.4)	
No	59 (57.3)	41 (58.6)	18 (54.5)	
Unknown	3 (2.9)	0 (0.0)	3 (9.1)	
Father on ART*** (*N*=41)				0.87**
Yes	21 (51.2)	14 (48.3)	7 (58.3)	
No	18 (43.9)	13 (44.8)	5 (41.7)	
Unknown	2 (4.9)	2 (6.9)	0 (0.0)	

ART: antiretroviral therapy; EART: early ART; PMTCT: prevention of mother-to child transmission; IQR: interquartile range

* *p*-value from Wilcoxon rank-sum test

** *p*-value from Fisher's exact test

*** self-reported.

At the initiation of ART, 62% of the children had already reached a WHO clinical Stage 3 or 4: this proportion was significantly higher in Ouagadougou than in Abidjan, 88% versus 48%, respectively (*p*<0.01). The median percentage of CD4 cells was 20% (IQR: 13.5 to 26.2) and 85% of children had moderate to severe immune deficiency at baseline. Children from Ouagadougou had a significantly lower CD4 percentage compared to those in Abidjan (*p*<0.01). Children included in Ouagadougou were also significantly more malnourished than those in Abidjan when considering all anthropometric indicators, with average weight-for-age, height-for-age and weight-for-height z-scores being significantly lower (*p*<0.01). The median haemoglobin level was 9.0 grams per decilitre (IQR: 8.2 to 9.9) and 80% of children had a haemoglobin level <10 grams per decilitre, with a proportion significantly higher in Ouagadougou than in Abidjan (*p*<0.01). The average viral load was 6 log10 (SD: 1.1), children from Ouagadougou having a significantly higher viral load compared to those in Abidjan (*p*<0.01) (Table 4).

**Table 4:** Baseline clinical, anthropometric, haematological, immunological and virological characteristics of the 161 HIV-infected children included in the ANRS 12206 MONOD study according to site (Abidjan, Ouagadougou, May 2011 to February 2013)

	Total *N=*161	Abidjan *n=*103	Ouagadougou *n=*58	
		
Characteristics	*n* (%)	*n* (%)	*n* (%)	*p*
WHO stage at ART initiation				<0.01
1	48 (29.8)	41 (39.8)	7 (12.1)	
2	13 (8.1)	13 (12.6)	0 (0.0)	
3	63 (39.1)	34 (33.0)	29 (50.0)	
4	37 (30.0)	15 (14.6)	22 (37.9)	
Weight-for-age z-score				
Mean (SD)	−2.4 (1.7)	−2.0 (1.6)	−3.1 (1.5)	<0.01
≥−2	75 (46.6)	61 (59.2)	14 (24.1)	<0.01
−2, −3	28 (17.4)	13 (12.6)	15 (25.9)	
<−3	58 (36.0)	29 (28.2)	29 (50.0)	
Height-for-age z-score				
Mean (SD)	−2.3 (1.8)	−1.9 (1.9)	−2.8 (1.6)	<0.01
≥−2	78 (48.5)	61 (59.2)	17 (29.3)	<0.01
−2, −3	34 (21.1)	17 (16.5)	17 (29.3)	
<−3	49 (30.4)	25 (24.3)	24 (41.4)	
Weight-for-height z-score				
Mean (SD)	−1.6 (1.5)	−1.3 (1.5)	−2.2 (1.5)	<0.01
≥−2	103 (64.0)	77 (74.8)	26 (44.9)	<0.01
−2, −3	30 (18.6)	15 (14.6)	15 (25.8)	
<−3	28 (17.4)	11 (10.6)	17 (29.3)	
Haemoglobin at initiation (g/dl)				
Median (IQR)	9.0 (8.2 to 9.9)	9.2 (8.5 to 10.0)	8.4 (7.8 to 9.5)	<0.01*
≥10	32 (19.9)	25 (24.2)	7 (12.1)	0.03**
9 to 10	48 (29.8)	34 (33.0)	14 (24.1)	
7 to 9	79 (49.1)	42 (40.8)	37 (63.8)	
Missing	2 (1.2)	2 (2.0)	0 (0.0)	
Median CD4 percentage at ART initiation (IQR)	19.5 (13.5 to 26.2)	21.5 (15.6 to 28.8)	16.6 (11.0 to 23.5)	<0.01*
Immune status at ART initiation				0.58
Normal	21 (13.0)	14 (13.6)	7 (12.1)	
Moderate	20 (12.5)	16 (15.5)	4 (6.9)	
Advanced	25 (15.5)	15 (14.6)	10 (17.2)	
Severe	92 (57.1)	56 (54.4)	36 (62.1)	
Missing	3 (1.9)	2 (1.9)	1 (1.7)	
Mean viral load at ART initiation (log10)	6.1 (1.1)	5.9 (1.1)	6.5 (0.9)	<0.01
Viral load class at ART initiation				0.02
≤6 log	64 (39.7)	48 (46.6)	16 (27.6)	
>6 log	95 (59.0)	53 (51.4)	42 (72.4)	
Missing	2 (1.3)	2 (2.0)	0 (0.0)	

SD: standard deviation; IQR: Interquartile range; ART: antiretroviral therapy; WHO: World Health Organization. Immune status: Age <11 months: normal CD4% >35%, moderate CD4% ≤35% and ≥30%, advanced CD4% ≤25% and ≥29%, severe CD4% <25%; Age ≥11 months: normal CD4% >30%, moderate CD4% ≤25% and ≥30%, advanced CD4% ≤20% and ≥24%, severe CD4% <20%.

* *p*-value from Wilcoxon rank-sum test

** *p*-value from Fisher's exact test

*** WHO 2006 growth standards.

## Discussion

Our study reported, for the first time, pre-EART attrition during the first 24 months of life in West African settings of intermediate HIV prevalence and in the context of very low coverage of early infant diagnosis (29% in Ouagadougou; 16% in Abidjan). The inclusion rate of children referred after a first positive DBS was 74%. The reasons for non-inclusion in the MONOD cohort included refusal of EART due to fear of stigmatization by the father, early competing infant mortality and false-positive HIV test on DBS. Good communication between parents, especially when the mother's and the child's HIV status are disclosed to the father, and being older than 12 months were two factors associated with a greater acceptability of EART in this research context. As a result of the low early infant diagnosis coverage in this context, children were diagnosed at a late age (median eight months of age), were referred at a median age of 12 months and subsequently initiated ART at an advanced age (median 14 months). The majority of the children were malnourished and started EART at an advanced clinical and immunological stage. These figures show a significant delay in Ouagadougou compared to Abidjan. In addition, we reported a limited access to PMTCT interventions, with more than half of the children included not having received any PMTCT intervention, despite this being recommended in these settings.

Of the 217 children referred to the MONOD study, 9% were found to be falsely HIV positive after the confirmatory test usually required before the initiation of EART. Indeed, the positive predictive value of this test depends on the prevalence of the disease in the tested population. As a result, in low HIV prevalence settings as in our context, the rate of false positives remains of concern and fully justifies the need for a systematic confirmatory test recommended by WHO [18]. According to an HIV transmission rate of 5%, the rate of false-positive DBS could reach 10.3% [19]. This finding confirms the need to verify the first HIV positive test result in the setting of intermediate HIV prevalence, but further highlights the need for lasting stringent quality control and confirmatory testing. In African settings, the long delay between the collection of the initial blood sample and the availability of the results after a confirmatory test complicates the disclosure of the initial positive result to the family, as well as the monitoring of the children over the cascade of care [20]. The problem of false-positive results can also undermine community trust in the reliability of early infant diagnosis and lead to possible diagnosis and EART delays. It also exposes HIV-uninfected children to the risk of antiretroviral side effects if they are not identified.

We show that EART is feasible in our context, reaching 77% of the children with a confirmed HIV diagnosis. However, despite the referral network set up to capture children eligible for EART through the MONOD project, the initiation of antiretroviral treatment was considerably delayed compared to the CHER strategy, especially in Burkina Faso, cumulating in delays in the pre-EART cascade (HIV diagnosis, HIV confirmation, EART initiation). The MONOD cohort aimed at initiating ART as soon as possible after early infant diagnosis (from 12 weeks). However, our findings showed that more than half of the children started EART after their first birthday, with high competing mortality before the initiation of EART. Children in Burkina Faso were older at the initiation of EART compared to those in Côte d'Ivoire. This difference between the study sites can be explained by the fact that there is a closer link between PMTCT services and postnatal services in Abidjan as compared to Ouagadougou. One of the healthcare centres of the MONOD project in Abidjan included a PMTCT project. Another likely explanation is the fact that the majority of children in Ouagadougou were recruited from the two university hospitals while they presented HIV-related symptoms, which led the paediatricians to propose an HIV diagnostic test for them. This late HIV infection testing at a symptomatic stage of illness has already been described elsewhere in Ethiopia [21]. Other studies have indicated that paediatric ART is often started too late, when children are already advanced in their disease and are immunodeficient [22–27]. This also occurred in the field reality of the MONOD settings and most likely explained the early high mortality rate, highlighting the importance of starting ART as early as possible.

The delay in the initiation of EART is essentially due to the difficulty of early infant diagnosis, as already observed in other studies, in low income countries [11,27,28]. This delay in access to early diagnosis can be explained by the lack of service delivery, especially because of the insufficient availability of virological diagnostics, the lack of reagents, the time required to perform tests, the lack of support services for the samples’ transportation between healthcare sites and laboratories, the lack of integration of testing within existing programmes such as immunization programmes, the missed opportunities to diagnose children in hospital and the low acceptability of early screening by the community and healthcare workers [11,12,29–32].

In our study, the cumulative rate of EART initiation before the age of two years reached 77%. Five studies conducted in Africa and Asia have reported proportions of ART initiation ranging between 40% and 99% [33]. This variability can be partly explained by the fact that the studies were performed at different timing regarding the subsequent recommendations for antiretroviral treatment initiation. In addition, these studies have included older children with a median age ranging between 2.2 and 6.5 years [33]. In our study, we report that variables related to the quality of communication within the parental couple regarding maternal and child HIV status disclosure proved to be important factors to favour an EART initiation. EART initiation also depends on several socio-economic factors such as the cost of transportation, availability of food aid, time constraint, perception of child health, stigma, beliefs and paternal support [34]. In our study, the cost of transportation was not considered as an obstacle as we provided transportation fees to the caregivers. The non-disclosure of the maternal and the child's HIV status to the father was clearly a missed opportunity to initiate EART. A child's HIV positive status usually indicates maternal infection and the resultant anxiety, fear of blame, social and healthcare discrimination as well as marital abandonment may negatively influence the mother's acceptance of the HIV status of the child [35,36]. Braitstein and al. showed that 30% of children's caregivers in Kenya reported that the child was lost-to-follow up because they did not want to share the child's HIV status with their partner [37].

We also found that being older than 12 months at the first contact visit increased the probability of initiation of EART. This result was likely due to the delay in HIV diagnosis, with testing often performed late, but also reflects the perception of the child's health by the parents. Indeed, parents agreed more readily to an HIV test and to initiation of therapy in children who were symptomatic [34]. Over one-third of children included in our study were already severely malnourished, with a higher proportion in Ouagadougou than in Abidjan. The WHO clinical stage and immunodeficiency status were also more advanced, consistently reflecting the recruitment bias in the hospital settings in Ouagadougou compared to Abidjan.

Our study also highlighted the limited access to PMTCT intervention in West African settings with an intermediate HIV prevalence, with more than 50% of children not having received any PMTCT prophylaxis. This situation is partly due to the missed opportunities of prenatal HIV testing. PMTCT interventions are proposed in a context where stigma remains high and where a significant number of pregnant women do not receive antenatal care. The conditions of access to prenatal and infant HIV diagnosis and antiretroviral treatment upstream of the MONOD project were discussed elsewhere [10]. These results demonstrated the difficulties in the implementation of PMTCT interventions in West Africa. The linkage between PMTCT and postnatal care services needs to be strengthened in order to identify HIV infection and initiate EART at the earliest opportunity [38]. It is urgent to raise awareness of both the community and healthcare workers in order to increase the number of mothers and children receiving PMTCT and achieve the goal of “zero new paediatric infections” [39].

Few cases of tuberculosis were identified in our settings (5/161; 3%), reflecting a difference of contextual morbidity in West Africa compared to South Africa [40]. Finally, our results highlight the delayed pre-ART cascade with a low coverage of EID mentioned in West Africa compared to South Africa [41].

Our study has limitations. First, our data represent only urban settings, probably reflecting a better access to early infant diagnosis and antiretroviral treatment compared to rural settings, where the delay of care could be even longer than that reported in our study. A few variables presented a high number of missing data (data on vital status of the father, his HIV status and antiretroviral treatment, which were recorded at the pre-inclusion visit). These missing data were mainly related to children whose parents did not accept the inclusion in the MONOD project or those who died before inclusion. Although that selection bias might have affected the precision of our estimate, we feel that it might have underestimated the OR estimates. Finally, the need to get either both parents’ consent (or the mother's if she was the only legal guardian, or the legal caregiver if parents were not alive) before inclusion of the children and EART initiation in this research context could have also underestimated the true EART acceptability rate. Those children who did not have parental consent were included in the ART programme but not in the research cohort. This may not depict the real life situation, but this allows us to highlight the place of the father in the family's interaction with the field reality and the mothers' difficulties of coping with a long-term daily ART strategy for themselves and their children without the father knowing it. Despite these limitations, our findings are original. This is the first study to document the pre-ART cascade and family acceptability of EART in children less than two years in the West African context.

## Conclusions

Competing early mortality of HIV-infected infants and fear of stigma within the parental couple play a key role in the missed opportunities of EART for children in West Africa. A public health effort must be made in order to take care of HIV-exposed infants and to identify HIV-infected infants earlier by strengthening linkages between PMTCT and paediatric HIV facilities, increasing community awareness on the need to treat children early, providing a global package of care for HIV-positive mothers (access to ART, child nutrition, improvement of socio-economic conditions), involving the father at the earliest opportunity according to maternal willingness, facilitating communication within couples about the disclosure of the child's HIV status and finally by fighting against stigma at the community level and among healthcare workers. These efforts are needed to provide early HIV care to infants and to improve their survival as efficiently as shown in the CHER trial [6]. More recently, in 2015, universal treatment of all HIV-infected patients was recommended by WHO [42]. However children less than two years old are those with the most advanced stage of the disease, requiring more rapid initiation of ART compared to older ones [43]. In the view of the currently enormous unmet need for paediatric ART provision compared to adults and the 2015 guidelines [42], it is necessary to provide ART for all children, but also as soon as possible for the youngest children, who are at a higher risk of death compared to older children.
